# Effects of Pinglu Canal Construction on Camouflage in Two Sesarmid Crab Species

**DOI:** 10.3390/biology15080651

**Published:** 2026-04-20

**Authors:** Binyu Zhai, Fuxi Lai, Sichen Lu, Xinglai Deng, Wenjie Li, Lin Yang, Changyin Deng, Zhihong Wu, Yiran Huang, Haitao Wang, Yuhao Liao, Rongping Bu

**Affiliations:** Guangxi Key Laboratory of Beibu Gulf Marine Biodiversity Conservation, College of Marine Sciences, Beibu Gulf University, Qinzhou 535011, China; zby020301@163.com (B.Z.); fuxilai47@163.com (F.L.); kiny1210moonlit@163.com (S.L.); x2768995685x@163.com (X.D.); 17607875690@163.com (W.L.); 18807863739@163.com (L.Y.); 19577049298@163.com (C.D.); 18328574954@163.com (Z.W.); 18507731978@163.com (Y.H.); 19177757917@163.com (H.W.); liaoyuhao131@163.com (Y.L.)

**Keywords:** camouflage, crab, background matching, disruptive coloration, Chromatic Just Noticeable Difference (CJND)

## Abstract

Canal construction activities alter the visual characteristics of natural habitats, which in turn impairs the camouflage effectiveness of resident animals. To explore the impacts of habitat degradation induced by anthropogenic engineering on the camouflage strategies of sympatric crab species, this study investigated the camouflage performance of *Chiromantes haematocheir* and *Orisarma dehaani* in natural estuarine habitats and canal-modified estuarine habitats of the Pinglu Canal. The Chromatic Just Noticeable Difference (CJND) of *C. haematocheir* in canal-modified habitats was 16.272 ± 9.503, significantly higher than the value of 12.911 ± 7.982 in natural estuaries (Z = −6.514, *p* < 0.001). Within the same habitat type, the CJND value of *O. dehaani* was consistently and significantly lower than that of *C. haematocheir*, with significant differences observed in both natural estuaries (Z = −11.572, *p* < 0.001) and canal-modified habitats (Z = −13.413, *p* < 0.001). Meanwhile, the pattern energy difference (PED) of *O. dehaani* in canal-modified habitats (0.744 ± 0.119) was significantly higher than that in natural estuaries (0.726 ± 0.107; Z = −2.390, *p* = 0.017), and the PED of *C. haematocheir* in canal-modified habitats (0.750 ± 0.133) was significantly higher than that in natural estuaries (0.731 ± 0.122; Z = −2.742, *p* = 0.006). The Pinglu Canal’s construction significantly exacerbated the reduction in background-matching camouflage effectiveness of both crab species. This study provides empirical evidence for evaluating the impacts of anthropogenic engineering activities on the camouflage adaptability of estuarine benthic animals, and offers a scientific reference for the conservation of benthic biological resources in canal-modified estuarine ecosystems.

## Simple Summary

Canal construction alters the natural visual characteristics of estuarine habitats, reducing the camouflage effectiveness of resident crabs and threatening their survival. We selected the estuary of the Pinglu Canal as our study area. This study compared camouflage performance between *Chiromantes haematocheir* (De Haan, 1883) and *Orisarma dehaani* (H. Milne Edwards, 1853) in natural and canal-modified estuaries and measured their key camouflage indices. In terms of the Chromatic Just Noticeable Difference, canal construction significantly reduced the background-matching effectiveness of both crab species, with *Chiromantes haematocheir* being more severely affected due to its bright body coloration. Neither species has curved carapace setae to attach to environmental materials for camouflage, nor can they rapidly change body color. Thus, they struggle to adapt to rapid habitat changes. This study reveals the negative impacts of human engineering activities on crab camouflage. The findings provide a scientific reference for the conservation of benthic animal resources in canal-modified estuarine ecosystems and offer empirical evidence for the assessment of the ecological impacts of similar water conservancy projects.

## 1. Introduction

Camouflage is a vital anti-predation strategy for animals, which involves modulating the visual traits (e.g., color, pattern, and morphology) of the body to reduce the probability of being detected and recognized by predators [1], thereby playing an indispensable role in the survival and reproduction of species [2,3]. Based on adaptive evolutionary mechanisms, camouflage is mainly classified into two primary types: background matching and disruptive coloration. Background matching refers to the ability of animals to align their body color, brightness, and texture with the substrate of their habitats, thereby minimizing their detectability by predators [1,2]. In contrast, disruptive coloration relies on high-contrast patterns on the body surface to break up the animal’s body contour, creating “false edges” or “visual segmentation” that hinder predators from recognizing the animal as a complete target [2,4]. Regardless of the camouflage type, its effectiveness is inherently dependent on the specific visual characteristics of the native habitat. When the habitat undergoes drastic anthropogenic or natural changes, animals with habitat-specific camouflage adaptations may fail to keep pace with the rapid environmental shifts, leading to a decline or even complete loss of camouflage efficacy [5,6].

In the Anthropocene, human activities have altered natural habitats at a rate far exceeding the pace of natural evolutionary processes [7], with river channel dredging and canal construction being typical forms of aquatic habitat disturbance. Canal construction fundamentally restructures the visual environment of riparian and estuarine zones through three primary mechanisms. First, it entails replacing natural sediment banks and vegetated shorelines with engineered hard materials such as concrete, thereby markedly diminishing the visual complexity of the habitat [8,9]. Concurrently, construction activities and associated riparian development elevate concentrations of suspended inorganic particulates within the water column, reducing light penetration and modifying the chromatic contrast between prey and underlying substrates [10,11,12]. Furthermore, the removal of riparian and fringing vegetation erodes textural heterogeneity and depletes the availability of natural camouflage materials, such as leaf litter and organic debris, thereby further undermining the potential for animal concealment [13]. These environmental changes directly threaten the survival of animals that rely on visual camouflage for anti-predation, especially species with limited mobility and relatively fixed camouflage strategies, as such changes may significantly increase their risk of predation [12,13].

**Figure 1:**
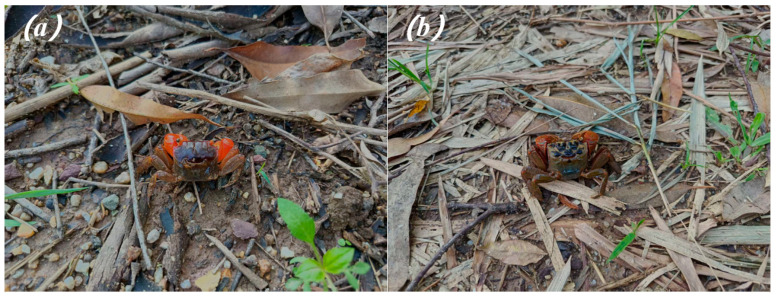
*Chiromantes haematocheir* (**a**) and *Orisarma dehaani* (**b**).

As a functionally significant taxonomic group within intertidal and estuarine ecosystems, crabs have been extensively studied in the context of phenotypic and environmental visual matching in camouflage [14,15,16,17]. For example, the carapace patterns of *Carcinus maenas* exhibit adaptive variation in response to different substrate types, facilitating phenotypic congruence with the surrounding habitat [14]. This species demonstrates a capacity for rapid physiological color adjustment, dispersing melanin on dark backgrounds and contracting pigment cells on light backgrounds to maintain brightness matching with the substrate [16]. Additionally, *C. maenas* engages in active background selection, choosing habitats that align with its intrinsic coloration to enhance concealment—a behavioral strategy that enables effective camouflage in heterogeneous environments (Figure 1) [15,17,18].

Although substantial progress has been made in understanding crab camouflage [19], empirical investigations into the direct effects of anthropogenic habitat modification on the camouflage efficacy of sympatric crab species remain limited [16]. *Chiromantes haematocheir* and *Orisarma dehaani* are congeneric species within the family Sesarmida and are dominant benthic macroinvertebrates in estuarine and mangrove ecosystems along the southern coast of China [20]. These two species display markedly different chromatic and patterning traits: *C. haematocheir* exhibits vivid, high-contrast body coloration, whereas *O. dehaani* possesses more cryptic coloration that closely resembles the spectral properties of natural estuarine substrates. Their sympatric distribution, combined with divergent camouflage-related phenotypes, renders them valuable model organisms for investigating the ecological implications of environmental change on animal concealment strategies. Birds serve as the primary predators of intertidal crabs, which is why we adopted an avian visual model to quantify camouflage performance. Previous research has demonstrated that evaluating crab camouflage effectiveness through the visual modeling of avian predators—particularly potential predators in intertidal systems—yields more sensitive and ecologically meaningful assessments [21]. Moreover, background matching at the microhabitat scale (<1 m^2^) has been identified as a critical determinant of camouflage success in benthic crab species [21].

In this study, we quantified the camouflage effectiveness of *C. haematocheir* and *O. dehaani* in the Pinglu Canal estuary, a typical anthropogenically modified aquatic ecosystem. We aimed to evaluate how visual environment changes in a habitat caused by canal construction affect the camouflage performance of sympatric crab species with distinct phenotypic traits. By comparing the differences in camouflage effectiveness between natural estuaries and canal-modified estuaries for both species, we aimed to reveal the adaptive responses of benthic crab camouflage to canal construction and provide scientific insights for conserving benthic faunal resources in canal-modified estuarine ecosystems.

## 2. Materials and Methods

### 2.1. Study Area

**Figure 2:**
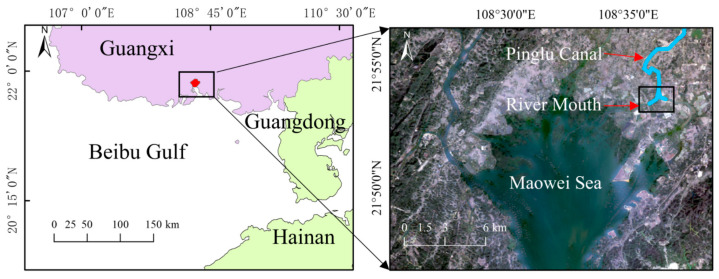
Estuarine sampling area for this study.

The Pinglu Canal, a major inter-basin waterway project in Guangxi, China, with its estuary located at the tip of Qinzhou Bay (Beibu Gulf), was chosen as the core study area (Figure 2). The specific study area is located within the channel range enclosed by the following four geographic coordinates: 108.59968496680747° E, 21.904191316158812° N; 108.61939704882218° E, 21.903638310440176° N; 108.60015427689301° E, 21.888388803378092° N; 108.61746433214299° E, 21.888074353976535° N. This area is undergoing drastic habitat restructuring due to large-scale canal construction activities (e.g., channel dredging, shoreline hardening, and bank revetment). The main anthropogenic changes to the habitat include the replacement of natural riverbanks with hard substrates such as concrete, a significant increase in water turbidity caused by construction-induced suspended solids, and the simplification and destruction of native vegetation structures. These anthropogenic disturbances have directly altered the key visual environmental characteristics of the estuary, including substrate color, texture complexity, light penetration, and background homogeneity. Therefore, this area is an ideal research setting to investigate the impacts of habitat modification on the camouflage effectiveness of benthic animals that rely on visual matching for anti-predation.

### 2.2. Sample Collection and Image Acquisition

Field sampling and image acquisition were conducted in the natural estuarine habitats and canal-modified estuarine habitats of the Pinglu Canal estuary from 10 April to 20 October 2024. Quadrat sampling and visual search were used to collect adult specimens of *C. haematocheir* and *O. dehaani*. We collected *n* = 18 adult *C. haematocheir* and *n* = 27 adult *O. dehaani* specimens (Table 1); all specimens were healthy and undamaged. There is no obvious intraspecific color variation in either species, so differences in sample size would not alter the analytical results. Individuals were collected by random sampling, and we did not deliberately control the sample size to be equal between the two species. The sample sizes employed here are consistent with those used in similar studies [16].

In situ photographs of the crabs and their native substrate backgrounds were taken immediately after collection, with the photographed substrate area being approximately 100 cm × 80 cm in size (matching the microscale for crab background matching [21]). To minimize variations in image quality caused by lighting conditions, all photographs were taken between 09:00 and 16:00 on sunny days, and a black sunshade was used to avoid shadows caused by direct sunlight. A Nikon D7000 digital camera equipped with a 105 mm macrolens (Nikon Corporation, Tokyo, Japan) was used for imaging. For human-visible light photography, a Baader UV/IR Cut filter (Baader Planetarium, Munich, Germany), which only transmits wavelengths between 400 and 700 nm, was mounted in front of the lens. For ultraviolet (UV) imaging, we used a Baader U-Venus filter (Baader Planetarium, Munich, Germany), transmitting wavelengths between 300 and 400 nm. Together, the lens and filters provided the following approximate overall sensitivity for each image channel, covering the UV and visible light bands that match the visual bandwidth of avian predators (the main predators of the two crab species): UV (300–400 nm), shortwave (SW, 400–550 nm), medium wave (MW, 420–620 nm), and long wave (LW, 560–700 nm).

For color calibration, each crab was photographed alongside standard reflectance cards (10% and 80% reflectance, covering 300–750 nm; JY-RD, Jingyi Optoelectronics, Guangzhou, China), which were placed at the center of the image to minimize color bias and enable standardized post-processing corrections. All images were saved in uncompressed RAW format with consistent camera settings (aperture, shutter speed, and ISO) throughout the sampling process. A tripod was used to keep the camera lens perpendicular to the sample area, and a black umbrella was used to ensure uniform and consistent lighting during shooting, with white balance calibration performed using the standard reflectance card before each shooting session. Finally, *n* = 20 images of *C. haematocheir* and *O. dehaani* in natural habitats, and *n* = 49 images of each species in canal-modified habitats, were obtained to subsequently analyze their camouflage effectiveness.

### 2.3. Image Preprocessing

To minimize illumination variations across different images, linear normalization correction was applied to all RAW images prior to image analysis, following the method of Troscianko and Stevens [22]. The ImageJ 1.53e software (Wayne Rasband and contributors, National Institutes of Health, Bethesda, MD, USA) was used for image processing, with the “Generate multispectral image” function employed to integrate UV and visible light images. Standard reflectance levels of 10% and 80% were set as calibration references, and the corresponding areas on the standard reflectance card were selected for each imported image to complete standardization correction and generate multispectral images [22,23]. All subsequent quantitative analyses of camouflage indices were performed on the calibrated multispectral images.

### 2.4. Quantification of Camouflage Indices

#### 2.4.1. Just Noticeable Difference

The image analysis functions in the ImageJ 1.53e software were used to analyze the brightness, chromaticity, and pattern energy differences between the crab carapace and the background substrate in the calibrated images. The Chromatic Just Noticeable Difference (CJND) and Luminance Just Noticeable Difference (LJND) were calculated to quantify chromatic and brightness matching, respectively, between the crabs and the substrate [22,24,25]. Since most avian predators of crabs are tetrachromats, possessing color vision based on four types of cone cells sensitive to LW, MW, SW, and UV light [26], a blue tit (*Cyanistes caeruleus*) visual model (a classic tetrachromatic visual model for avian predators [27]) was used to evaluate the chromatic and brightness differences between the crabs and the substrate in this study. A smaller JND value indicates a higher degree of matching between the crab and the background, thus representing superior camouflage effectiveness [27].

In the custom ImageJ toolkit, multispectral images were converted to cone catch values, with a natural light illumination system (D65) and a Wyszecki coefficient of 0.02 used for the conversion [28]. This setup enabled the quantitative analysis of the chromaticity, luminance, and pattern energy of the selected image regions (crab carapace and background substrate) [22]. The chromaticity and luminance of the selected regions were calculated with a starting pixel size of 2 and an ending pixel size of 512 [29], and the pixel increment per step was set to 1.414 times (exponential growth) [29]. Luminance measurements were scaled from 0 (minimum brightness) to 65,535 (maximum brightness), covering the full brightness range of 32 bit TIFF images [30].

#### 2.4.2. Pattern Energy Difference

The pattern energy difference (PED) was calculated to quantify the pattern matching between the crab carapace and the background substrate [22,25]. The PED reflects the difference in texture and pattern energy between the crab and the substrate, with a smaller PED value indicating a higher degree of pattern matching and better camouflage effectiveness [22]. The PED was calculated using the same calibrated multispectral images and ImageJ toolkit used for CJND and LJND, with the same selection of image regions and pixel size parameters [22,29].

#### 2.4.3. Gabor Edge Disruption Ratio

The Gabor edge disruption ratio (GabRat) was computed to quantify the efficacy of disruptive coloration in crabs [22,31]. GabRat quantifies the proportion of false edges relative to true edges along the carapace contour, reflecting the extent to which internal patterning disrupts the integrity of the true carapace boundary [31]. GabRat values are bounded between 0 and 1, with higher values denoting greater interference of the true contour by internal false edges, indicating enhanced concealment of the carapace margin within the background substrate. Conversely, lower values correspond to more conspicuous true edges and consequently reduced disruptive effectiveness [31]. According to the classification criteria established by Troscianko et al. [31], GabRat values exceeding 0.2 suggest a measurable degree of edge blending, whereas values greater than 0.4 are indicative of highly effective disruptive coloration.

The GabRat tool in the ImageJ 1.53e software was used for the measurement [22,31]. First, calibrated multispectral images were converted into binary mask images, and the true edges of the crab carapace were manually selected. Gabor filters were then applied at different angles to each pixel in the target region (crab carapace) and each pixel along the true edge of the carapace [31,32]. The filter parameters and measurement angles were set based on the ratio of image pixels to the actual scale. GabRat values were randomly measured at five distinct locations on the carapace within the substrate background, and the average of these five measurements was taken as the final GabRat value for the crab in that habitat.

### 2.5. Scanning Electron Microscopy (SEM) of Crab Carapaces

To determine whether the setae on the carapace surface of the two crab species could serve as a medium for attaching environmental materials (a common structural camouflage strategy for many crabs [12,33]), scanning electron microscopy was used to observe the morphological characteristics of the carapace setae. Healthy adult crabs (12–45 mm CW) of both species were collected, and small pieces of carapace tissue (5 mm × 5 mm) were excised from the dorsal surface of the carapace. Tissue samples were desiccated to a constant mass, sputter-coated with gold, and subsequently examined and imaged via scanning electron microscopy at a standardized magnification of 500×. The sputter coating was performed using a Quorum SC7620 Sputter Coater (Quorum Technologies, Laughton, UK), and SEM imaging was performed using a TESCAN MIRA LMS scanning electron microscope (TESCAN ORSAY HOLDING, Brno, Czech Republic) equipped with an Oxford Instruments EDS detector (Oxford Instruments, High Wycombe, UK). For both species, the presence or absence of curved setae—the primary morphological structures implicated in the adherence of environmental materials [12,33]—was documented along the carapace surface.

### 2.6. Statistical Analysis

All statistical analyses were performed using the IBM SPSS Statistics 27.0 software (IBM Corp., Armonk, NY, USA). Prior to statistical analysis, we used the Shapiro–Wilk test to determine the normality of the data (CJND, LJND, PED, and GabRat) and Levene’s test to determine the homogeneity of variance. Since the data did not conform to a normal distribution, the non-parametric Mann–Whitney U test was used to compare the differences in CJND, LJND, PED, and GabRat values between *C. haematocheir* and *O. dehaani* in the same habitat type, and between the same species in natural and canal-modified habitats. A significance level of *p* < 0.05 was used for all statistical tests, and all data were expressed as the mean ± standard deviation (mean ± SD).

## 3. Results

All original measurement data used in this study are provided in File S1.

### 3.1. Morphological Characteristics of Crab Carapace Setae

**Figure 3:**
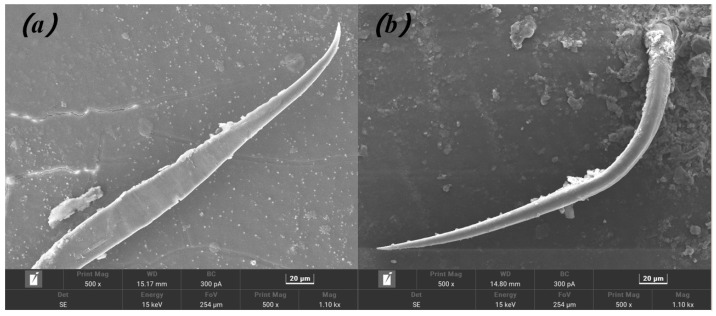
Scanning electron micrographs of *Chiromantes haematocheir* (**a**) and *O. dehaani* (**b**) (magnification: 500×).

Scanning electron microscopy (SEM) observations revealed that both *C. haematocheir* and *O. dehaani* lacked curved setae on their carapaces; only smooth, straight setae were present on the dorsal carapace surface of both species (Figure 3). No morphological structures suitable for attaching environmental materials were found on the carapaces of either species.

### 3.2. Differences in Camouflage Indices Between the Two Crab Species

#### 3.2.1. Chromatic Just Noticeable Difference (CJND)

**Figure 4:**
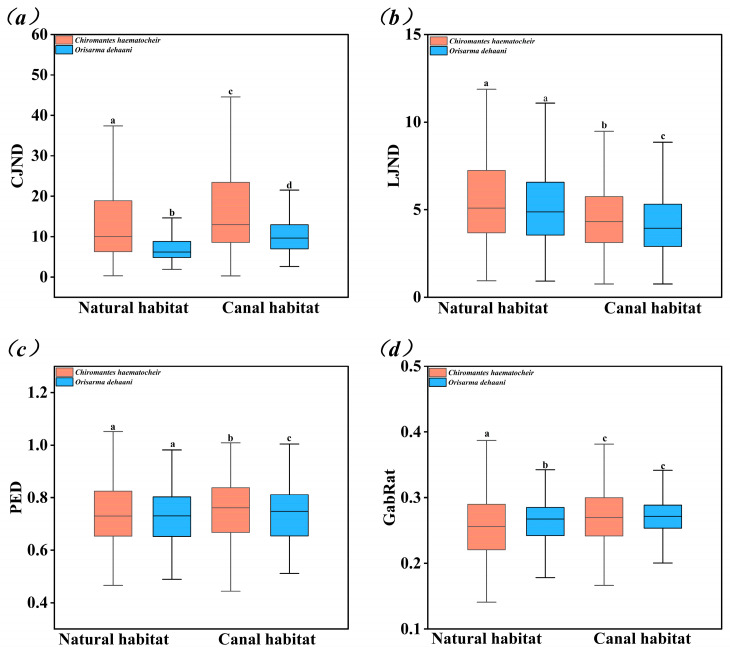
Comparison of camouflage indices between *Chiromantes haematocheir* and *O. dehaani* in natural and canal-modified estuarine habitats. (**a**) Chromatic Just Noticeable Difference (CJND), (**b**) Luminance Just Noticeable Difference (LJND), (**c**) pattern energy difference (PED), and (**d**) Gabor edge disruption ratio (GabRat). Different letters indicate significant differences between groups (*p* < 0.05); same letters indicate no significant difference (*p* > 0.05).

Significant differences in CJND values were observed between the two crab species and between different habitat types (Figure 4a, *p* < 0.001). In natural estuaries, the CJND value of *O. dehaani* (7.102 ± 3.137) was significantly lower than that of *C. haematocheir* (12.911 ± 7.982, Z = −11.572, *p* < 0.001). In canal-modified habitats, the CJND value of *O. dehaani* (10.729 ± 5.396) was also significantly lower than that of *C. haematocheir* (16.272 ± 9.503, Z = −13.413, *p* < 0.001). The CJND value of *O. dehaani* in canal-modified habitats was significantly higher than in natural estuaries (Z = −16.313, *p* < 0.001), and the same trend was observed for *C. haematocheir* (Z = −6.514, *p* < 0.001).

#### 3.2.2. Luminance Just Noticeable Difference (LJND)

The LJND values showed distinct variation patterns between habitat types and between the two crab species (Figure 4b). In natural estuaries, no significant difference in LJND values was found between *O. dehaani* (5.457 ± 2.990) and *C. haematocheir* (6.236 ± 4.329, Z = −1.850, *p* = 0.064). In contrast, in canal-modified habitats, the LJND value of *O. dehaani* (4.311 ± 1.993) was significantly lower than that of *C. haematocheir* (4.684 ± 2.317, Z = −3.846, *p* < 0.001). The LJND value of *O. dehaani* in canal-modified habitats was significantly lower than in natural estuaries (Z = −8.544, *p* < 0.001), and the LJND value of *C. haematocheir* also showed a significant decrease in canal-modified habitats (Z = −6.105, *p* < 0.001).

#### 3.2.3. Pattern Energy Difference (PED)

The PED values of both crab species were significantly higher in canal-modified habitats than in natural estuaries, with interspecific differences only observed in canal-modified habitats (Figure 4c). In natural estuaries, no significant difference in PED values was found between *O. dehaani* (0.726 ± 0.107) and *C. haematocheir* (0.731 ± 0.122, Z = −0.534, *p* = 0.593). In canal-modified habitats, the PED value of *O. dehaani* (0.744 ± 0.119) was significantly lower than that of *C. haematocheir* (0.750 ± 0.133, Z = −2.405, *p* = 0.016). For the same species, the PED value of *O. dehaani* in canal-modified habitats was significantly higher than in natural estuaries (Z = −2.390, *p* = 0.017), and the PED value of *C. haematocheir* also showed a significant increase in canal-modified habitats (Z = −2.742, *p* = 0.006).

#### 3.2.4. Gabor Edge Disruption Ratio (GabRat)

The GabRat values of both species were significantly higher in canal-modified habitats than in natural estuaries, with interspecific differences only observed in natural estuaries (Figure 4d). In natural estuaries, the GabRat value of *O. dehaani* (0.264 ± 0.040) was significantly higher than that of *C. haematocheir* (0.256 ± 0.051, Z = −2.311, *p* = 0.021). In canal-modified habitats, no significant difference in GabRat values was found between *O. dehaani* (0.272 ± 0.034) and *C. haematocheir* (0.272 ± 0.051, Z = −0.805, *p* = 0.421). The GabRat value of *O. dehaani* in canal-modified habitats was significantly higher than in natural estuaries (Z = −4.504, *p* < 0.001), and the GabRat value of *C. haematocheir* also increased significantly in canal-modified habitats (Z = −4.544, *p* < 0.001). Notably, the GabRat values of both species were below 0.4 in both habitat types, failing to reach the threshold for high blending rate (effective disruptive coloration).

## 4. Discussion

### 4.1. Impacts of Canal Construction on the Background Matching of the Two Crab Species

In natural estuarine habitats, *O. dehaani* exhibited significantly lower CJND values than *C. haematocheir*, indicating superior chromatic matching to natural substrates—a finding consistent with its cryptic coloration that closely resembles native mud–sand sediments. By contrast, the vivid, high-contrast carapace patterning of *C. haematocheir* resulted in greater chromatic disparity with the background, yielding elevated CJND values [21,34]. Canal construction significantly increased CJND values in both species, demonstrating that anthropogenic habitat modification diminishes chromatic background-matching effectiveness. This finding corroborates previous work [21] reporting that the structural heterogeneity of natural estuarine substrates facilitates optimal camouflage, whereas the homogenized, hard substrates characteristic of canal-modified environments impair chromatic congruency between crabs and their background.

The disproportionately greater increase in CJND observed for *C. haematocheir* in modified habitats suggests that this species experiences more severe impairment of chromatic matching following canal construction. This heightened susceptibility likely stems from its conspicuous coloration, which may be maintained by sexual selection [15,35]; in particular, against the uniformly light, featureless, hard substrates of canal-modified habitats, such pigmentation renders *C. haematocheir* more detectable to avian predators, thereby increasing predation risk and directly threatening its survival [36]. In contrast, the comparatively cryptic coloration of *O. dehaani* more closely resembles modified substrates, resulting in a more modest CJND elevation and the retention of relatively effective chromatic matching.

The significant reduction in luminance contrast (LJND) observed in both species within canal-modified habitats is primarily attributable to elevated water turbidity resulting from construction and dredging activities. Suspended fine sediments and deposited silt increase the overall brightness of the substrate surface [10], whereas natural estuarine substrates—such as organic detritus and mud—exhibit inherently lower reflectance. Furthermore, the microtopographic complexity of undisturbed substrates generates localized shading, producing measurable luminance disparity between crabs and their surroundings [3]. When background substrates become uniformly brighter in modified habitats due to sediment deposition and reduced water clarity, the relative brightness differential between crabs and the background diminishes, accounting for the observed decline in the LJND.

The significant increase in pattern energy difference (PED) for both species in canal-modified habitats provides additional evidence that anthropogenic modification disrupts pattern matching with the background substrate [22], consistent with the patterns observed for chromatic matching (CJND). Interspecific divergence in the PED, evident only in modified habitats, reflects differential adaptive responses in pattern-based camouflage between sympatric species exposed to environmental alteration. Such variation in camouflage traits may confer community-level anti-predator benefits [37]: avian predators, characterized by limited brain volume and a lack of specialization on crab prey, exhibit reduced capacity to efficiently process interference from heteromorphic prey phenotypes [38]. Consequently, despite diminished individual-level pattern matching in modified habitats, the coexistence of species exhibiting divergent PED values may sustain partial protective effects for crab assemblage [38,39]. Moreover, under conditions constraining predator foraging decisions, even suboptimal pattern camouflage can reduce detection risk [39,40], potentially mitigating the fitness consequences of reduced pattern matching for both species in canal-modified environments.

### 4.2. Impacts of Canal Construction on the Disruptive Coloration of the Two Crab Species

The GabRat values for both *C. haematocheir* and *O. dehaani* remained below the 0.4 threshold in both natural and canal-modified habitats, indicating that neither species achieved the level of high blending efficacy associated with effective disruptive coloration [31]. This suggests that disruptive coloration does not constitute a primary anti-predation camouflage strategy for either species in these environmental contexts. Although canal construction significantly increased GabRat values in both species, the observed values consistently fell below 0.4, implying that the enhancement of disruptive effectiveness remains biologically inconsequential and insufficient to offset the diminished background-matching efficacy resulting from chromatic and pattern mismatches.

In natural estuarine habitats, the numerous burrows excavated by crabs can also interfere with the rapid identification of crabs by avian predators by potentially causing a distraction, thus serving as a supplementary anti-predation strategy [41]. This camouflage-enhancing mechanism has been verified in other invertebrates; for example, *Allenaltica flavicornis* creates numerous leaf-like holes on foliage while feeding, which reduces its detectability by avian predators [41]. The two crab species considered in this study can quickly retreat into the burrows upon detecting predators, providing valuable concealment time even with suboptimal disruptive coloration [36]. However, canal construction has destroyed the natural sediment substrate of estuaries and replaced it with hard concrete, significantly reducing the number of crab burrows and thus weakening this supplementary anti-predation strategy, further increasing the survival pressure of the two crab species in canal-modified habitats.

Notably, despite remaining below 0.4, the observed increase in GabRat values in canal-modified habitats may reflect a temporal lag in ecological response following canal construction. As the time since habitat alteration increases, GabRat values could continue to rise and gradually reach a new adaptive equilibrium as crabs adjust their disruptive camouflage strategies to the long-term modified visual environment.

### 4.3. Morphological Constraints on the Camouflage Adaptation of the Two Crab Species

Scanning electron microscopy confirmed that both *C. haematocheir* and *O. dehaani* lack curved setae on their carapaces, which is a key morphological constraint on their camouflage adaptation to canal construction. Decorator crabs (*Majoidea*) can attach environmental materials to their curved setae to achieve structural camouflage [12,33]; however, the two species in this study do not have curved setae and thus lack this ability. In addition, neither species can modify their body color to adapt to the rapid changes in habitat appearance caused by canal construction.

The two species in this study can only rely on their fixed body coloration and pattern traits for camouflage, which further diminishes their camouflage effectiveness in canal-modified habitats [12,35]. Morphological constraints appear to have a more significant impact on *C. haematocheir*; in particular, its vivid, fixed body coloration is highly mismatched with the homogeneous substrate of canal estuaries, and its lack of structural and behavioral camouflage strategies makes it unable to compensate for this mismatch. In contrast, the body coloration of *O. dehaani* is more similar to the modified substrate, so morphological constraints have a relatively smaller impact on its camouflage effectiveness.

Furthermore, the bright red claws of *C. haematocheir* may have evolved as a sexual signaling trait, and in natural habitats, this trait may also divert predator attention from the body to the claws, thereby enhancing survival odds [3]. However, this strategy becomes counterproductive in the homogeneous estuarine environment of the Pinglu Canal: the contrast between the bright red claws and the dull carapace is significantly heightened against the homogeneous hard substrate, making the claws more easily detectable by avian predators [16]. The dull carapace also fails to blend with the modified background, resulting in a “double mismatch” of the claws and carapace with the substrate and further increasing the detection risk of *C. haematocheir* [16]. In contrast, *O. dehaani* has no such bright sexual signaling traits, and its body coloration is uniformly matched with the substrate, thus avoiding this counterproductive anti-predation strategy and exhibiting greater survival stability in canal-modified habitats [42]. This is reflected in the lower population abundance of *Chiromantes haematocheir* (18 individuals) compared to *Orisarma dehaani* (27 individuals) at this site.

## 5. Conclusions

This study demonstrates that canal construction significantly altered the estuarine visual environment—through substrate homogenization, increased turbidity, and vegetation loss—thereby reducing background-matching camouflage (chromatic and pattern) in both species. Additionally, both species lack morphological (curved setae) and physiological (rapid color change) adaptations for dynamic camouflage, rendering them particularly vulnerable to habitat modification. These findings underscore the importance of maintaining visual heterogeneity in estuarine habitats for benthic crabs reliant on camouflage. Future research should examine long-term population dynamics and adaptive potential under sustained anthropogenic disturbance.

## Figures and Tables

**Table 1:** Number of photographs per habitat and per species.

Item	Number
Natural estuary	20
Canal estuary	49
*Chiromantes haematocheir*	17
*Orisarma dehaani*	27

## Data Availability

The data presented in this study are available in the Supplementary Materials.

## Supplementary Materials

The following supporting information can be downloaded at: https://www.mdpi.com/article/10.3390/biology15080651/s1, File S1. Raw_Data.

## Abbreviations

The following abbreviations are used in this manuscript:

CJND	Chromatic Just Noticeable Difference
LJND	Luminance Just Noticeable Difference
PED	Pattern Energy Difference
GabRat	Gabor Edge Disruption Ratio
SEM	Scanning Electron Microscopy
UV	Ultraviolet
SW	Shortwave
MW	Medium Wave
LW	Long Wave
GenAI	Generative Artificial Intelligence
APC	Article Processing Charge
